# On the Performance of Random Cognitive mmWave Sensor Networks

**DOI:** 10.3390/s19143184

**Published:** 2019-07-19

**Authors:** Yi Song, Weiwei Yang, Zhongwu Xiang, Biao Wang, Yueming Cai

**Affiliations:** 1 School of Physics and Electronic Electrical Engineering, Huaiyin Normal University, Huai’an 223300, China; 2 College of Communications Engineering, Army Engineering University of PLA, No. 88 Houbiaoying, Qinhuai District, Nanjing 210007, China; 3 Jiangsu Province Key Construction Laboratory of Modern Measurement Technology and Intelligent System, Huai’an 223300, China; 4 School of Electronic and Information, Jiangsu University of Science and Technology, Zhenjiang 212003, China

**Keywords:** cognitive radio, millimeter wave, physical layer security, secrecy throughput

## Abstract

This paper investigates the secrecy performance of a cognitive millimeter wave (mmWave) wiretap sensor network, where the secondary transmitter (SU-Tx) intends to communicate with a secondary sensor node under the interference temperature constraint of the primary sensor node. We consider that the random-location eavesdroppers may reside in the signal beam of the secondary network, so that confidential information can still be intercepted. Also, the interference to the primary network is one of the critical issues when the signal beam of the secondary network is aligned with the primary sensor node. Key features of mmWave networks, such as large number of antennas, variable propagation law and sensitivity to blockages, are taken into consideration. Moreover, an eavesdropper-exclusion sector guard zone around SU-Tx is introduced to improve the secrecy performance of the secondary network. By using stochastic geometry, closed-form expression for secrecy throughput (ST) achieved by the secondary sensor node is obtained to investigate secrecy performance. We also carry out the asymptotic analysis to facilitate the performance evaluation in the high transmit power region. Numerical results demonstrate that the interference temperature constraint of the primary sensor node enables us to balance secrecy performance of the secondary network, and provides interesting insights into how the system performance of the secondary network that is influenced by various system parameters: eavesdropper density, antenna gain and sector guard zone radius. Furthermore, blockages are beneficial to improve ST of the secondary sensor node under certain conditions.

## 1. Introduction

Future communication will probably realize man–machine–object cooperative communication and ultra-densely connections to achieve full coverage, and higher spectral efficiency will become a key issue. Owing to the spectrum scarcity problem and the increasing data rate demand with different quality of service (QoS), new spectrum bands are needed to meet explosive traffic requirements. In order to improve spectrum utilization efficiency and avoid interference with other operational networks, it is necessary to enable cognitive radio characteristics in densely deployed future networks. On the other hand, as a promising candidate for future mobile networks, millimeter-wave (mmWave) communication [1] has attracted more and more attention because of its operating frequency ranging from 30 to 300 GHz. One of the significant features of mmWave signals is the small wavelength, which allows the transceivers to deploy large numbers of antenna arrays to achieve beamforming gain to compensate path loss and establish links with a reasonable signal-to-interference-plus-noise ratio (SINR).

More recently, researchers have conducted some studies on cognitive mmWave networks, most of which focus on various techniques to dynamically avoid interference and lessen mutual interference. In order to minimize the mutual interference between cognitive users and other operational networks in fifth-generation (5G) networks, wavelet packet transform was applied to sparse code multiple access systems for spectrum sensing and multi-user access modulation in [2]. Using a similar method, [3] studied a joint wavelet-based spectrum sensing and filter bank multicarrier modulation for cognitive 5G heterogeneous networks in a mmWave spectrum band. Additionally, [4] showed that in mmWave cellular networks with a shared spectrum, especially when sharing spectrum with a high-power operator and density, it was necessary for inter-operator base station coordination to repress the resulting cross-operator interference. However, malicious devices may partake in spectrum sensing and access due to the open and dynamic characteristics of cognitive radio, which makes a cognitive radio more vulnerable to malicious attacks and eavesdropping [5]. For example, malicious attackers may mislead legitimate cognitive users and even cause them to fail to work properly by imitating the primary user sending information to the secondary user and informing the secondary user that the authorized spectrum has been occupied. Compared with malicious attacks, passive eavesdropping is more difficult to detect by users, resulting in more serious security threats. As a result, new security challenges from all aspects of network architecture are faced by a cognitive radio, such as spectrum sensing, spectrum access and spectrum management [6].

Physical layer security (PLS) attempts to protect wireless networks from wiretapping by utilizing randomness of wireless medium at the physical layer [7,8]. Therefore, PLS has been identified as a promising strategy to implement secure communication, which has received extensive attention in research community [9,10,11,12,13,14,15]. To elaborate, PLS has been considered in cognitive networks [16,17,18]. Specifically, for the cognitive networks with a random distribution of eavesdropping nodes, the authors designed four different transmission schemes to achieve secure transmission under the interference constraint of the primary receiver [16]. Also, for random cognitive radio networks, a simple and decentralized secure transmission scheme combining the secrecy guard zone and artificial noise was proposed to enhance the secure information transmission of secondary networks [17]. Moreover, the authors investigated the PLS of a multi-user multi-eavesdropper cognitive radio system [18]. Furthermore, for dual phase amplify-and-forward large wireless sensor networks, a new security cooperative protocol was studied [19].

On the other hand, PLS in mmWave systems has aroused interest with enthusiasm [20,21,22,23]. The features of the mmWave communication system, such as large antenna array, directionality and short range transmission, may provide stronger PLS for mmWave system. Using analog beamforming in the mmWave base station, the authors analyzed the secrecy throughput from the perspectives of delay-limited and delay-tolerant transmissions [24]. Under the stochastic geometry framework, the secrecy performance was studied in the artificial-noise-assisted and the noise-limited mmWave networks [25], the network-wide PLS performance of the downlink transmission in an mmWave cellular network was comprehensively studied. Considering a large-scale mmWave ad hoc network, the authors examined the impact of artificial noise on the secrecy rate; the results showed that it was necessary to carefully determine the power allocation between artificial noise and information signals to improve secrecy performance [26]. In addition, combined with unmanned aerial vehicle (UAV) networks, the secure transmission of mmWave simultaneous wireless information and power transfer UAV relay networks was studied [27]. Considering unique characteristics of air-to-ground channels and practical constraints of UAV deployment, [28] studied the secrecy performance of millimeter-wave unmanned aerial vehicle networks.

All the above works were either focused on the PLS of traditional microwave cognitive wireless networks or on the analysis of the secrecy performance of mmWave networks, but were rarely investigated on the PLS for cognitive mmWave wireless networks. In addition, as mentioned earlier, future communications will probably be ultra-densely heterogeneous networks, where communication networks can be built with a combination of macro cells and small cells. The existing macro cells guarantee coverage and mobility in the ultra high frequency band, while small cells are needed in order to achieve gigabit rates over the mmWave range of 30–300 GHz. Effective utilization of small cell spectrum bands is one of the ways to improve the obtainable capacity and data rate of heterogeneous networks. It is a challenging task that could be accomplished through the spectrum sensing capability of cognitive radio [2]. Therefore, the design of future communication networks requires us to embed cognitive features including the ability of sensing interference power to take advantage of the available spectrum bands. Furthermore, because of the remarkable characteristics of the mmWave channel, for instance, the mmWave signals are more sensitive to blocking effects, and the fading statistics of the line-of-sight (LOS) link and the non-line-of-sight (NLOS) link are completely different [29], the secrecy performance of cognitive mmWave sensor networks will be very different from that of traditional cognitive microwave networks, which needs to be re-evaluated. Besides, considering that random-location eavesdroppers may reside in the signal beam of the secondary network, so that confidential information can still be intercepted. Also, the interference with the primary network is one of the critical issues when the signal beam of the secondary network is aligned with the primary network. To the best of our knowledge, the research on the secrecy performance of cognitive mmWave wiretap sensor networks has not yet been addressed, which motivates our work.

This paper studies the PLS in cognitive mmWave wiretap sensor networks. Our analysis considers the key characteristics of the mmWave channel and blockage density and the effects of different antenna arrays gains. In particular, the main contributions of this paper in specific terms are

- By using stochastic geometry approaches, we model the cognitive mmWave wiretap sensor networks to characterize the random spatial locations of primary and secondary nodes, as well as the eavesdroppers. Taking into account the effect of blockage, the links are either LOS or NLOS. A sector secrecy guard zone, synonymously referred to as the eavesdropper-exclusion area, is invoked around the secondary transmitter (SU-Tx) for improving the PLS.
- Considering that the random-location of both eavesdroppers and the primary sensor node may reside in the signal beam of the secondary network, we derive analytical expressions of secrecy throughput (ST) for the secondary sensor node to measure secrecy performance of the system with arbitrary system parameters. Subsequently, an asymptotic expression is proposed to gain additional insight into the performance evaluation and design for the proposed system.
- The results show that the interference temperature constraint of the primary sensor node can be used to balance secrecy performance of the secondary network, which indicates losing the interference temperature constraint of the primary node would worsen secrecy performance. Increasing the radius of the sector guard zone can improve ST. Furthermore, it is also shown that increasing the transmit power or antenna gain of the secondary network does not necessarily lead to the improvement of ST, and sometimes deteriorate ST. Besides, blockages can also be used to improve ST of SU-Rx under certain conditions.

The rest of the paper is organized as follows. The system model and mmWave channel characteristics are provided in Section 2. Secrecy performance analysis is evaluated in Section 3. Then, in Section 4, numerical and simulation results are given. Finally, conclusions are drawn in Section 5.

## 2. System Model

### 2.1. Network Topology

**Figure 1:**
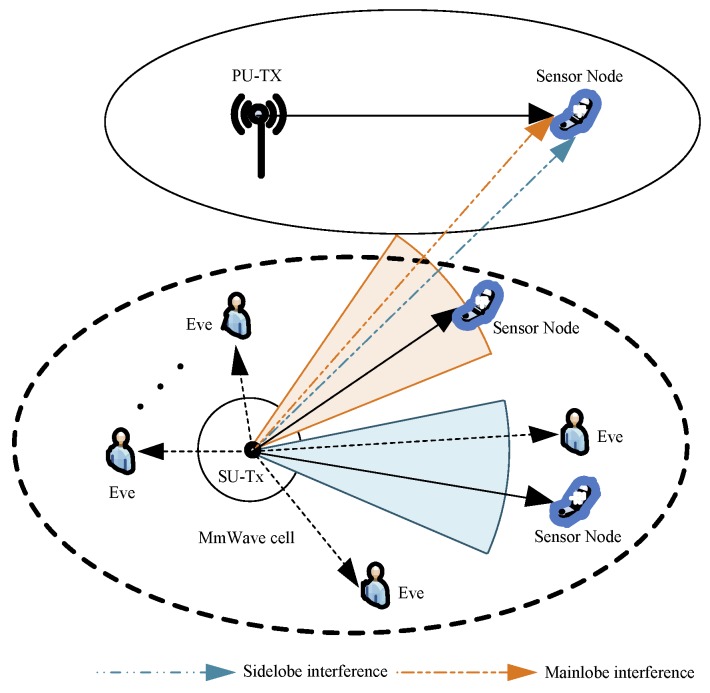
Illustration of the system model.

As shown in Figure 1, we consider an underlay cognitive mmWave wiretap sensor network, where the SU-Tx is equipped multiple antennas to utilize directional beamforming to communicate with a secondary sensor node in the presence of a primary sensor node. Multiple eavesdroppers intercept confidential information sent from the SU-Tx. The spatial distribution of all eavesdroppers is modeled using a homogeneous Poisson point processes (HPPP), which is denoted by $\Phi_{E}$ and it is associated with the density $\lambda_{E}$. Also, the locations of primary and secondary sensor nodes are randomly distributed, and both of them and each eavesdropper is equipped with one receive antenna.

### 2.2. Directional Beamforming

For mathematical tractability, the antenna pattern is assumed to be a sectorial model [25,26,30]. Consequently, the SU-Tx antenna gain pattern about a generic angle θ is given by

(1) $$G_{S}\left(\theta \right)=\left\{\begin{array}{ll} M_{S}, & \mathrm{if}\left|\theta \right|\leq \theta_{S}\\ m_{S}, & \mathrm{Otherwise} \end{array},\right.$$

where $M_{S}$ and $m_{S}$ are the main lobe and side lobe gain, respectively; $\theta \in \left[0,2\pi \right)$ is the angle of boresight direction; $\theta_{S}$ is the main lobe beam width. Assuming that the perfect channel state information (CSI) of the secondary sensor node can be obtained by the SU-Tx, then it could adjust its antenna steering orientation array to the secondary sensor node to maximize the directional gain. It should be pointed out that we neglect the channel estimation error and also ignore the error of time and carrier frequency synchronization. In practical, it may be a nontrivial task to estimate the CSI, an upper bound on achievable secrecy performance is actually provided in our work. The estimation of mmWave channel is more in line with the actual communication system, but it is beyond the scope of this paper. Furthermore, supposing that the eavesdroppers can be detected as long as they are close enough to SU-Tx. Therefore, a sector guard zone with central angle $\theta_{S}$ and radius *r* around the SU-Tx is introduced, where eavesdroppers are not allowed to roam. Similar security mechanisms have been used in the literature [22,31,32].

### 2.3. Channel Model

In outdoor mmWave scenarios, a channel between SU-Tx and receiver may be LOS or NLOS link in the presence of blockages [26]. $P_{L}\left(r_{d}\right)$ represents the probability of a LOS with distance $r_{d}$, while the NLOS probability is $P_{N}\left(r_{d}\right)$, which is given as $P_{L}\left(r_{d}\right)=e^{-\beta r_{d}}$ or $P_{N}\left(r_{d}\right)=1-e^{-\beta r_{d}}$, where β is a parameter determined by the density of blockages. Considering independent Nakagami fading for each link [33], and the Nakagami fading parameter of the LOS (NLOS) link is $N_{L}$($N_{N}$). For simplicity, $N_{L}$ and $N_{N}$ are both assumed to be positive integers. The secondary sensor node and eavesdroppers received channel gains can be described as $M_{S}{\left|h_{SU}\right|}^{2}L\left(r_{SU}\right)$ and $M_{S}{\left|h_{SE}\right|}^{2}L\left(r_{E}\right)$, respectively, where both ${\left|h_{SU}\right|}^{2}$ and ${\left|h_{SE}\right|}^{2}$ are normalized Gamma random variable with following ΓNL,11NLNL and ΓNN,11NNNN, the distance from the SU-Tx to the secondary sensor node is $r_{SU}$ and the distance from the SU-Tx to the eavesdroppers is $r_{E}$. The path loss function of $L\left(r_{SU}\right)$ and $L\left(r_{E}\right)$ are modeled as $L\left(r_{j}\right)=C_{L}r^{-\alpha_{L}}$ or $L\left(r_{j}\right)=C_{N}r^{-\alpha_{N}}$, $j\in \left\{SU,E\right\}$, $\alpha_{L}$ and $\alpha_{N}$ are path loss exponents of the LOS and NLOS, $C_{L}$ and $C_{N}$ are the constant depending on the LOS and NLOS.

### 2.4. Received SINR

To guarantee the interference temperature constraint of the primary network, the instantaneous interference power at the primary sensor node from the SU-Tx should lower than a given threshold $I_{w}$, then the transmit power of the SU-Tx is further constrained by $P_{U}=\min\left(\frac{I_{w}}{M_{S}{\left|h_{SP}\right|}^{2}L\left(r_{SP}\right)},P_{t}\right)$, where $P_{t}$ is the maximum transmit power of the SU-Tx. Moreover, it is assumed that the interference of primary transmitter on the secondary sensor node is not greater than $\delta_{0}$. The variable $\delta_{0}$ leaves for future research. $\sigma_{\vartheta }^{2},\vartheta \in \left\{U,E\right\}$ is the noise power. Therefore, the SINR at the secondary sensor node is defined as

(2) $$\gamma_{U}=\frac{M_{S}P_{U}{\left|h_{SU}\right|}^{2}L\left(r_{U}\right)}{\sigma_{U}^{2}+\delta_{0}},$$

In this model, the eavesdroppers attempt to intercept the confidential information of the system, assuming that the SU-Tx do not know the instantaneous CSI of eavesdroppers. Non-colluding eavesdroppers are taken into account where eavesdroppers decode the information independently. For such case, the eavesdropper closest to SU-Tx is not necessarily the most detrimental, but the eavesdropper with the best channel to SU-Tx. In addition, we consider the worst-case scenario of cognitive mmWave wiretap sensor networks, in which the eavesdroppers are assumed to have powerful detection capabilities. In fact, this assumption overestimates the anti-jamming ability of the eavesdroppers. Then, the instantaneous SINR of detecting the information of the secondary sensor node at the most detrimental eavesdropper can be expressed as follows:

(3) $$\gamma_{E}=\max_{E\in \phi_{E}}\left(\frac{M_{S}P_{U}{\left|h_{SE}\right|}^{2}L\left(r_{E}\right)}{\sigma_{E}^{2}}\right).$$

Of particular note, we consider that the primary and secondary sensor nodes and eavesdroppers are located in the main lobe. This is because the main lobe has the greatest interference to the primary sensor node, which may cause the primary network performance to be deteriorated seriously. At the same time, the situation where the eavesdropper is located in the main lobe poses a serious threat to the security of the secondary sensor network. Thus, the considered scenario is the worst-case which have been widely adopted in mmWave systems with secrecy considerations; e.g., [25,34].

## 3. Secrecy Performance Analysis

In this section, we elaborate to evaluate the ST of secondary sensor node for the proposed cognitive mmWave wiretap sensor networks.

### 3.1. ST of the Secondary Sensor Node

The ST of the secondary sensor node can be expressed as

(4) $$\begin{array}{ll} \eta^{U} & =R_{m}^{s}\mathrm{Pr}\left(\gamma_{E}<2^{\left(R_{m}-R_{m}^{s}\right)}-1,\gamma_{U}>2^{R_{m}}-1\right)\\ & =R_{m}^{s}\mathrm{Pr}\;\;\left(\max_{E\in \phi_{E}}\left(\;\;\frac{M_{S}P_{U}{\left|h_{SE}\right|}^{2}L\left(r_{E}\right)}{\sigma_{E}^{2}}\right)\;\;<\;\varepsilon_{2},\frac{P_{U}M_{S}{\left|h_{SU}\right|}^{2}L\left(r_{U}\right)}{\sigma_{U}^{2}+\delta_{0}}\;\;>\;\varepsilon_{1}\;\;\right) \end{array},$$

where $\varepsilon_{1}=2^{R_{m}}-1$, $\varepsilon_{2}=2^{\left(R_{m}-R_{m}^{s}\right)}-1$. Substituting $P_{U}$ in Equation (4) and using the properties of the joint distribution of two random variables in [35] we have:

(5) $$\begin{array}{l} \min\left(\frac{I_{w}}{M_{S}{\left|h_{SP}\right|}^{2}L\left(r_{SP}\right)},P_{t}\right)=\left\{\begin{array}{l} \frac{I_{w}}{M_{S}{\left|h_{SP}\right|}^{2}L\left(r_{SP}\right)}\;\mathrm{if}\;P_{t}>\frac{I_{w}}{M_{S}{\left|h_{SP}\right|}^{2}L\left(r_{SP}\right)}\\ \;\;\;\;P_{t}\;\;\;\;\mathrm{if}\;P_{t}\leq \frac{I_{w}}{M_{S}{\left|h_{SP}\right|}^{2}L\left(r_{SP}\right)} \end{array}\right. \end{array}.$$

Therefore, further derivation of Equation (4) can be expressed as

(6) $$\begin{array}{ll} \eta^{U} & =R_{m}^{s}\mathrm{Pr}\left(\gamma_{E}<2^{\left(R_{m}-R_{m}^{s}\right)}-1,\gamma_{U}>2^{R_{m}}-1\right)\\ & =R_{m}^{s}\mathrm{Pr}\left(\max_{E\in \phi_{E}}\left(\frac{M_{S}P_{t}{\left|h_{SE}\right|}^{2}L\left(r_{E}\right)}{\sigma_{E}^{2}}\right)<\varepsilon_{2},\frac{P_{t}M_{S}{\left|h_{SU}\right|}^{2}L\left(r_{U}\right)}{\sigma_{U}^{2}+\delta_{0}}>\varepsilon_{1},P_{t}\leq \frac{I_{w}}{M_{S}{\left|h_{SP}\right|}^{2}L\left(r_{SP}\right)}\right)\;\\ & +R_{m}^{s}\mathrm{Pr}\left(\max_{E\in \phi_{E}}\left(\frac{\frac{I_{w}}{{\left|h_{SP}\right|}^{2}L\left(r_{SP}\right)}{\left|h_{SE}\right|}^{2}L\left(r_{E}\right)}{\sigma_{E}^{2}}\right)<\varepsilon_{2},\frac{\frac{I_{w}}{{\left|h_{SP}\right|}^{2}L\left(r_{SP}\right)}{\left|h_{SU}\right|}^{2}L\left(r_{U}\right)}{\sigma_{U}^{2}+\delta_{0}}>\varepsilon_{1},P_{t}>\frac{I_{w}}{M_{S}{\left|h_{SP}\right|}^{2}L\left(r_{SP}\right)}\right)\;\\ & =R_{m}^{s}\underset{Q_{1}}{\underset{︸}{\int_{0}^{\frac{I_{w}}{P_{t}M_{S}}}\mathrm{Pr}\left(\max_{E\in \phi_{E}}\left(\frac{M_{S}P_{t}{\left|h_{SE}\right|}^{2}L\left(r_{E}\right)}{\sigma_{E}^{2}}\right)<\varepsilon_{2},\frac{P_{t}M_{S}{\left|h_{SU}\right|}^{2}L\left(r_{U}\right)}{\sigma_{U}^{2}+\delta_{0}}>\varepsilon_{1}\right)f_{k}\left(x\right)dx}}\;\\ & +R_{m}^{s}\underset{Q_{2}}{\underset{︸}{\int_{\frac{I_{w}}{P_{t}M_{S}}}^{\infty }\mathrm{Pr}\left(\max_{E\in \phi_{E}}\left(\frac{\frac{I_{w}}{{\left|h_{SP}\right|}^{2}L\left(r_{SP}\right)}{\left|h_{SE}\right|}^{2}L\left(r_{E}\right)}{\sigma_{E}^{2}}\right)<\varepsilon_{2},\frac{\frac{I_{w}}{{\left|h_{SP}\right|}^{2}L\left(r_{SP}\right)}{\left|h_{SU}\right|}^{2}L\left(r_{U}\right)}{\sigma_{U}^{2}+\delta_{0}}>\varepsilon_{1}\right)f_{k}\left(x\right)dx}} \end{array},$$

where $\kappa ={\left|h_{SP}\right|}^{2}L\left(r_{SP}\right)$, and the probability density function (PDF) of κ is represented by $f_{\kappa }\left(x\right)$, the cumulative distribution function (CDF) of κ is represented by $F_{\kappa }\left(x\right)$. We first derive the CDF of κ, which can be expressed as

(7) $$\begin{array}{ll} F_{k}\left(x\right) & =\mathrm{Pr}\left({\left|h_{SP}\right|}^{2}L\left(r_{SP}\right)<x\right)=\mathrm{Pr}\left({\left|h_{SP}\right|}^{2}<\frac{x}{L\left(r_{SP}\right)}\right)\;\\ & =\frac{2}{R_{SP}^{2}}\left(\int_{0}^{R_{SP}}\frac{\Upsilon \left(N_{L},\frac{xN_{L}}{L\left(r_{SP}\right)}\right)}{\Gamma \left(N_{L}\right)}e^{-\beta r_{SP}}r_{SP}dr_{SP}+\int_{0}^{R_{SP}}\frac{\Upsilon \left(N_{N},\frac{xN_{N}}{L\left(r_{SP}\right)}\right)}{\Gamma \left(N_{N}\right)}\left(1-e^{-\beta r_{SP}}\right)r_{SP}dr_{SP}\right)\;\\ & =\frac{2}{R_{SP}^{2}}\left(\sum_{i=0}^{\infty }\frac{{\left(-1\right)}^{i}{\left(\frac{N_{L}x}{C_{L}}\right)}^{N_{L}+i}}{i!\left(N_{L}+i\right)\Gamma \left(N_{L}\right)}\;\;\times \;\;\frac{\Upsilon \left(\alpha_{L}\left(N_{L}+i\right)+2,\beta R_{SP}\right)}{\beta^{\alpha_{L}\left(N_{L}+i\right)+2}}\right.\;\;+\;\;\left.\sum_{j=0}^{\infty }\;\;\frac{{\left(-1\right)}^{j}{\left(\frac{N_{N}x}{C_{N}}\right)}^{N_{N}+j}}{j!\left(N_{N}+j\right)\Gamma \left(N_{N}\right)}\left.\;\;\left(\;\;\frac{{\left(R_{SP}\right)}^{\alpha_{N}\left(N_{N}+j\right)+2}}{\alpha_{N}\left(N_{N}+j\right)+2}\;\;-\;\;\frac{\Upsilon \left(\alpha_{N}\left(N_{N}+j\right)+2,\beta R_{SP}\right)}{\beta^{\alpha_{N}\left(N_{N}+j\right)+2}}\right.\;\;\right)\;\;\right) \end{array}.$$

Then, we solve the first derivative of CDF and obtain the PDF of κ, which is calculated as

(8) $$\begin{array}{ll} f_{k}\left(x\right) & =\;\;\frac{2}{R_{SP}^{2}}\;\;\left(\;\;\sum_{i=0}^{\infty }\frac{{\left(-1\right)}^{i}{\left(x\right)}^{N_{L}+i-1}}{{\left(\frac{C_{L}}{N_{L}}\right)}^{N_{L}+i}i!\Gamma \left(N_{L}\right)}\;\;\times \;\;\frac{\Upsilon \left(\alpha_{L}\left(N_{L}+i\right)+2,\beta R_{SP}\right)}{\beta^{\alpha_{L}\left(N_{L}+i\right)+2}}\right.\;\;\;\\ & +\;\;\left.\sum_{j=0}^{\infty }\;\;\frac{{\left(-1\right)}^{j}{\left(x\right)}^{N_{N}+j-1}}{{\left(\frac{C_{N}}{N_{N}}\right)}^{N_{N}+j}j!\Gamma \left(N_{N}\right)}\left.\;\;\left(\;\;\frac{{\left(R_{SP}\right)}^{\alpha_{N}\left(N_{N}+j\right)+2}}{\alpha_{N}\left(N_{N}+j\right)+2}\;\;-\;\;\frac{\Upsilon \left(\alpha_{N}\left(N_{N}+j\right)+2,\beta R_{SP}\right)}{\beta^{\alpha_{N}\left(N_{N}+j\right)+2}}\right.\right)\right) \end{array}.$$

Now, we focus our attention on deriving the integrals $Q_{1}$ in Equation (6). $Q_{1}$ can be rewritten as

(9) $$\begin{array}{l} Q_{1}=\int_{0}^{\frac{I_{w}}{P_{t}M_{S}}}F_{y}\left(\varepsilon_{2}\right)\left(1-F_{v}\left(\varepsilon_{1}\right)\right)f_{k}\left(x\right)dx, \end{array}$$

where

(10) $$\begin{array}{ll} F_{y}\left(\varepsilon_{2}\right) & =\mathrm{Pr}\left\{\max_{E\in \phi_{E}}\left(\frac{M_{S}P{\left|h_{SE}\right|}^{2}L\left(r_{E}\right)}{\sigma_{E}^{2}}\right)<\varepsilon_{2}\right\}\\ & =\underset{\Psi_{1}}{\underset{︸}{\mathrm{Pr}\left\{\max_{E\in \phi_{{}_{E}}^{L}}\left(\frac{M_{S}P_{t}{\left|h_{SE}\right|}^{2}L\left(r_{E}\right)}{\sigma_{E}^{2}}\right)<\varepsilon_{2}\right\}}}\times \underset{\Psi_{2}}{\underset{︸}{\mathrm{Pr}\left\{\max_{E\in \phi_{{}_{E}}^{N}}\left(\frac{M_{S}P_{t}{\left|h_{SE}\right|}^{2}L\left(r_{E}\right)}{\sigma_{E}^{2}}\right)<\varepsilon_{2}\right\}}}, \end{array}$$

(11) $$\begin{array}{l} F_{v}\left(\varepsilon_{1}\right)=\mathrm{Pr}\left\{\frac{P_{t}M_{S}{\left|h_{SU}\right|}^{2}L\left(r_{U}\right)}{\sigma_{U}^{2}+\delta_{0}}<\varepsilon_{1}\right\}. \end{array}$$

$F_{y}\left(\varepsilon_{2}\right)$ and $F_{v}\left(\varepsilon_{1}\right)$ can be obtained after some mathematical manipulations. However, for the sake of completeness, we presented a sketch of the proof, as given in Appendix A.

Whereas, we can get $Q_{1}$ in Equation (6), which can be expressed as

(12) $$\begin{array}{ll} Q_{1} & =\left(1-\frac{2}{R_{U}^{2}}\left(\sum_{i=0}^{\infty }\frac{{\left(-1\right)}^{i}{\left(A\varepsilon_{1}N_{L}\right)}^{N_{L}+i}}{i!\left(N_{L}+i\right)\Gamma \left(N_{L}\right){\left(P_{t}M_{S}C_{L}\right)}^{N_{L}+i}}\times \frac{\Upsilon \left(\alpha_{L}\left(N_{L}+i\right)+2,\beta R_{U}\right)}{\beta^{\alpha_{L}\left(N_{L}+i\right)+2}}\right.\right.\;\\ & +\left.\left.\sum_{j=0}^{\infty }\frac{{\left(-1\right)}^{j}{\left(A\varepsilon_{1}N_{N}\right)}^{N_{N}+j}}{j!\left(N_{N}+j\right)\Gamma \left(N_{N}\right){\left(P_{t}M_{S}C_{N}\right)}^{N_{N}+j}}\left.\left(\frac{{\left(R_{U}\right)}^{\alpha_{N}\left(N_{N}+j\right)+2}}{\alpha_{N}\left(N_{N}+j\right)+2}-\frac{\Upsilon \left(\alpha_{N}\left(N_{N}+j\right)+2,\beta R_{U}\right)}{\beta^{\alpha_{N}\left(N_{N}+j\right)+2}}\right.\right)\right)\right)\;\\ & \times \frac{2}{R_{SP}^{2}}\left(\sum_{i=0}^{\infty }\frac{{\left(-1\right)}^{i}{\left(N_{L}I_{w}\right)}^{N_{L}+i}}{i!\left(N_{L}+i\right)\Gamma \left(N_{L}\right){\left(P_{t}M_{S}\right)}^{N_{L}+i}}\times \frac{\Upsilon \left(\alpha_{L}\left(N_{L}+i\right)+2,\beta R_{SP}\right)}{\beta^{\alpha_{L}\left(N_{L}+i\right)+2}}\right.\;\\ & +\left.\sum_{j=0}^{\infty }\frac{{\left(-1\right)}^{j}{\left(N_{N}I_{w}\right)}^{N_{N}+j}}{j!\left(N_{N}+j\right)\Gamma \left(N_{N}\right){\left(P_{t}M_{S}\right)}^{N_{N}+i}}\left.\left(\frac{{\left(R_{SP}\right)}^{\alpha_{N}\left(N_{N}+j\right)+2}}{\alpha_{N}\left(N_{N}+j\right)+2}-\frac{\Upsilon \left(\alpha_{N}\left(N_{N}+j\right)+2,\beta R_{SP}\right)}{\beta^{\alpha_{N}\left(N_{N}+j\right)+2}}\right.\right)\right)\\ & \times \exp\left(-\theta_{s}\lambda_{E}\left(\left(\frac{\left(N_{N}-1\right)!}{\Gamma \left(N_{N}\right)}\right)\sum_{m=0}^{N_{N}-1}\frac{{\left(\frac{\sigma_{E}^{2}\varepsilon_{2}N_{N}}{P_{t}C_{N}M_{S}}\right)}^{m}}{m!}\times \frac{\Gamma \left(\frac{m\alpha_{N}+2}{\alpha_{N}},\frac{\sigma_{E}^{2}\varepsilon_{2}r^{\alpha_{N}}N_{N}}{P_{t}C_{N}M_{S}}\right)}{\alpha_{N}{\left(\frac{\sigma_{E}^{2}\varepsilon_{2}N_{N}}{P_{t}C_{N}M_{S}}\right)}^{\frac{m\alpha_{N}+2}{\alpha_{N}}}}\right.\right.\;\\ & +\left.\left.\sum_{n=0}^{\infty }\frac{{\left(-1\right)}^{n}{\left(\frac{\sigma_{E}^{2}\varepsilon_{2}N_{N}}{P_{t}C_{N}M_{S}}\right)}^{N_{N}+n}}{n!\left(N_{N}+n\right)\Gamma \left(N_{N}\right)}\frac{\Gamma \left(\alpha_{N}\left(N_{N}+n\right)+2,\beta r\right)}{\beta^{\alpha_{N}\left(N_{N}+n\right)+2}}-\sum_{n=0}^{\infty }\frac{{\left(-1\right)}^{n}{\left(\frac{\sigma_{E}^{2}\varepsilon_{2}N_{L}}{P_{t}C_{L}M_{S}}\right)}^{N_{L}+n}}{n!\left(N_{L}+n\right)\Gamma \left(N_{L}\right)}\frac{\Gamma \left(\alpha_{L}\left(N_{L}+n\right)+2,\beta r\right)}{\beta^{\alpha_{L}\left(N_{L}+n\right)+2}}\right)\right) \end{array}.$$

From the derivation of $Q_{1}$, we can see that when the radius of sector guard zone becomes larger or the SU-Tx uses a narrower beams, $Q_{1}$ increases. When $P_{t}\leq \frac{I_{w}}{M_{S}{\left|h_{SP}\right|}^{2}L\left(r_{SP}\right)}$, the larger $R_{SP}$ is beneficial to the increasing of $Q_{1}$. In addition, it can be seen from the expression of $Q_{1}$ that it is related to $\lambda_{E}$ and the blockage density β.

Let us now turn our attention to solving the integral $Q_{2}$ in Equation (6). Then $Q_{2}$ can be rewritten as

(13) $$\begin{array}{l} Q_{2}=\int_{\frac{I_{w}}{P_{t}M_{S}}}^{\infty }F_{z}\left(\varepsilon_{2}\right)\left(1-F_{u}\left(\varepsilon_{1}\right)\right)f_{k}\left(x\right)dx, \end{array}$$

where

(14) $$\begin{array}{ll} F_{z}\left(\varepsilon_{2}\right) & =\mathrm{Pr}\left\{\max_{E\in \phi_{E}}\left(\frac{\frac{I_{w}}{{\left|h_{SP}\right|}^{2}L\left(r_{SP}\right)}{\left|h_{SE}\right|}^{2}L\left(r_{E}\right)}{\sigma_{E}^{2}}\right)<\varepsilon_{2}\right\}\\ & =\underset{\Psi_{3}}{\underset{︸}{\mathrm{Pr}\left\{\max_{E\in \phi_{{}_{E}}^{L}}\left(\frac{I_{w}{\left|h_{SE}\right|}^{2}L\left(r_{E}\right)}{{\left|h_{SP}\right|}^{2}L\left(r_{SP}\right)\sigma_{E}^{2}}\right)<\varepsilon_{2}\right\}}}\times \underset{\Psi_{4}}{\underset{︸}{\mathrm{Pr}\left\{\max_{E\in \phi_{{}_{E}}^{N}}\left(\frac{I_{w}{\left|h_{SE}\right|}^{2}L\left(r_{E}\right)}{{\left|h_{SP}\right|}^{2}L\left(r_{SP}\right)\sigma_{E}^{2}}\right)<\varepsilon_{2}\right\}}}, \end{array}$$

(15) $$\begin{array}{l} F_{u}\left(\varepsilon_{1}\right)=\mathrm{Pr}\left\{\frac{I_{w}{\left|h_{SU}\right|}^{2}L\left(r_{U}\right)}{A{\left|h_{SP}\right|}^{2}L\left(r_{SP}\right)}<\varepsilon_{1}\right\}. \end{array}$$

$F_{z}\left(\varepsilon_{2}\right)$ and $F_{u}\left(\varepsilon_{1}\right)$ can be obtained after some mathematical manipulations. A sketch of this proof is given in Appendix B.

Consequently, we have $Q_{2}$, which can be expressed as

(16) $$\begin{array}{ll} Q_{2} & =\int_{\frac{I_{w}}{P_{t}M_{S}}}^{\infty }(1-\frac{2}{R_{U}^{2}}\left(\sum_{i=0}^{\infty }\frac{{\left(-1\right)}^{i}{\left(Ax\varepsilon_{1}N_{L}\right)}^{N_{L}+i}}{i!\left(N_{L}+i\right)\Gamma \left(N_{L}\right){\left(I_{w}C_{L}\right)}^{N_{L}+i}}\times \frac{\Upsilon \left(\alpha_{L}\left(N_{L}+i\right)+2,\beta R_{U}\right)}{\beta^{\alpha_{L}\left(N_{L}+i\right)+2}}\right.\\ & +\left.\sum_{j=0}^{\infty }\frac{{\left(-1\right)}^{j}{\left(Ax\varepsilon_{1}N_{N}\right)}^{N_{N}+j}}{j!\left(N_{N}+j\right)\Gamma \left(N_{N}\right){\left(I_{w}C_{N}\right)}^{N_{N}+i}}\left.\left(\frac{{\left(R_{U}\right)}^{\alpha_{N}\left(N_{N}+j\right)+2}}{\alpha_{N}\left(N_{N}+j\right)+2}-\frac{\Upsilon \left(\alpha_{N}\left(N_{N}+j\right)+2,\beta R_{U}\right)}{\beta^{\alpha_{N}\left(N_{N}+j\right)+2}}\right.\right)\right)\\ & \times \exp\left(-\right.\theta_{s}\lambda_{E}\left(\left(\frac{\left(N_{N}-1\right)!}{\Gamma \left(N_{N}\right)}\right)\sum_{m=0}^{N_{N}-1}\frac{{\left(\frac{\sigma_{E}^{2}\varepsilon_{2}xN_{N}}{I_{w}C_{N}}\right)}^{m}}{m!}\times \frac{\Gamma \left(\frac{m\alpha_{N}+2}{\alpha_{N}},\frac{\sigma_{E}^{2}\varepsilon_{2}xr^{\alpha_{N}}N_{N}}{I_{w}C_{N}}\right)}{\alpha_{N}{\left(\frac{\sigma_{E}^{2}\varepsilon_{2}xN_{N}}{I_{w}C_{N}}\right)}^{\frac{m\alpha_{N}+2}{\alpha_{N}}}}\right.\\ & +\left.\left.\sum_{n=0}^{\infty }\frac{{\left(-1\right)}^{n}{\left(\frac{\sigma_{E}^{2}\varepsilon_{2}xN_{N}}{I_{w}C_{N}}\right)}^{N_{N}+n}}{n!\left(N_{N}+n\right)\Gamma \left(N_{N}\right)}\frac{\Gamma \left(\alpha_{N}\left(N_{N}+n\right)+2,\beta r\right)}{\beta^{\alpha_{N}\left(N_{N}+n\right)+2}}-\sum_{n=0}^{\infty }\frac{{\left(-1\right)}^{n}{\left(\frac{\sigma_{E}^{2}\varepsilon_{2}xN_{L}}{I_{w}C_{L}}\right)}^{N_{L}+n}}{n!\left(N_{L}+n\right)\Gamma \left(N_{L}\right)}\frac{\Gamma \left(\alpha_{L}\left(N_{L}+n\right)+2,\beta r\right)}{\beta^{\alpha_{L}\left(N_{L}+n\right)+2}}\right)\right)\\ & \times \frac{2}{R_{SP}^{2}}\left(\sum_{i_{1}=0}^{\infty }\frac{{\left(-1\right)}^{i_{1}}{\left(N_{L}\right)}^{N_{L}+i_{1}}{\left(x\right)}^{N_{L}+i_{1}-1}}{i_{1}!\Gamma \left(N_{L}\right)}\times \frac{\Upsilon \left(\alpha_{L}\left(N_{L}+i_{1}\right)+2,\beta R_{SP}\right)}{\beta^{\alpha_{L}\left(N_{L}+i_{1}\right)+2}}\right.\\ & +\left.\sum_{j_{1}=0}^{\infty }\frac{{\left(-1\right)}^{j_{1}}{\left(N_{N}\right)}^{N_{N}+j_{1}}{\left(x\right)}^{N_{N}+j_{1}-1}}{j_{1}!\Gamma \left(N_{N}\right)}\left.\left(\frac{{\left(R_{SP}\right)}^{\alpha_{N}\left(N_{N}+j_{1}\right)+2}}{\alpha_{N}\left(N_{N}+j_{1}\right)+2}-\frac{\Upsilon \left(\alpha_{N}\left(N_{N}+j_{1}\right)+2,\beta R_{SP}\right)}{\beta^{\alpha_{N}\left(N_{N}+j_{1}\right)+2}}\right.\right)\right)dx \end{array}.$$

From Equation (16), we can know that the transmit power $P_{t}$, the main beam gain $M_{S}$, the the blockage density β and eavesdrop density $\lambda_{E}$ have an important effects on $Q_{2}$.

Resultantly, the calculation for the ST of the secondary sensor node is complete. Then, $\eta^{U}$ can be calculated as follows:

(17) $$\begin{array}{l} \eta^{U}=R_{m}^{s}\left(Q_{1}+Q_{2}\right), \end{array}$$

where $Q_{1}$ can be obtained from Equation (12) and $Q_{2}$ follows from the Equation (16).

**Remark** **1.** *According to Equation (17), $\eta^{U}$ is an increasing function due to the increasing of r, which demonstrates that the secrecy performance of secondary network is improved as r increases. Increasing antenna gain and $P_{t}$ are beneficial to the improvement of $\eta^{U}$, but could increase the risk of information leakage. In addition, the value of $I_{w}$ has a great impact on the secrecy performance of secondary sensor node. Therefore, according to the actual network environment, it should be carefully designed to achieve better performance of a secondary network. Furthermore, $\eta^{U}$ has a close relationship with $\lambda_{E}$, $R_{SP}$ and the blockage density β*.

### 3.2. Asymptotic Behavior

To extract additional insights about cognitive mmWave sensor wiretap networks, we derive the asymptotic expression of the ST with $P_{t}\to \infty$. By using the expression of Equation (17), the asymptotic behavior of ST can be easily calculated, that is, only $Q_{2}$ exists in Equation (17), and the lower limit of integration in $Q_{2}$ is changed to 0. From the asymptotic behavior of ST, we have found that increasing $I_{w}$ is not always conducive to the improvement of $\eta^{U}$, and excessive $I_{w}$ increases the risk of eavesdroppers eavesdropping confidential information. Therefore, the interference temperature constraint of the primary network enables a compromise between reliability and security of the cognitive mmWave sensor wiretap networks.

## 4. Numerical Results

Here, numerical results of the proposed cognitive mmWave wiretap networks are presented to evaluate its performance. The thermal noise power is assumed to be −90 dBm, and considering an mmWave system with carrier frequency at 28 GHz. As pointed out by [36], the parameters of Nakagami fading of the LOS and NLOS link are $N_{L}=3$ and $N_{N}=2$, and the path-loss model: $\beta_{L}$ = 61.4 dB, $\alpha_{L}=2$, $\beta_{N}$ = 72 dB, $\alpha_{N}=2.92$, $C_{L}=10^{-\frac{\beta_{L}}{10}}$ and $C_{N}=10^{-\frac{\beta_{N}}{10}}$. BPCU is short for bit per channel use.

**Figure 2:**
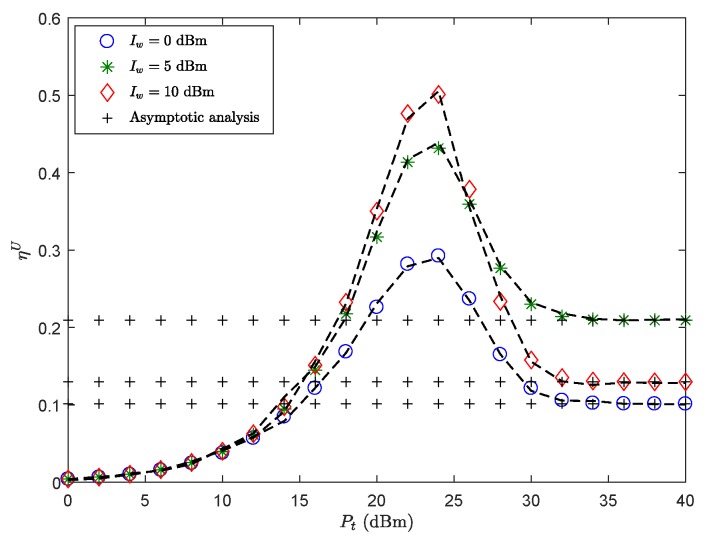
$\eta^{U}$ versus $P_{t}$ with $\lambda_{E}=0.001$ $nodes/m^{2}$, *r* = 60 m, $\theta_{S}=\frac{\pi }{3}$, $R_{m}=4.5$ BPCU, $R_{m}^{s}=1$ BPCU, β = $\frac{1}{141.4}$, $\delta_{0}=0.1$, $m_{S}=0.1$ and $M_{S}=200$.

Figure 2 plots $\eta^{U}$ of the secondary sensor node versus $P_{t}$ with different $I_{w}$. The results show that: (1) $P_{t}$ is not the larger the better. That is, larger $P_{t}$ would be more conducive to signal transmission, but worse secrecy performance. (2) The ST is close to the floor when $P_{t}$ is sufficiently high, in this case, the fixed interference power $I_{w}$ limits $P_{t}$ of the SU-Tx to affect ST. Interestingly, the ST in the high power region has a very low floor when $I_{w}$ is either extremely low or very higher. This can be explained as follows. When $I_{w}$ is lower, the adverse effects of reliability outweigh the benefits of security brought about by $I_{w}$. When $I_{w}$ is higher, the security problems caused by $I_{w}$ become the main deterioration factor of ST performance. Therefore, it is very important to choose the appropriate $I_{w}$ to weigh secrecy performance of the system. Furthermore, the ST first increases and then decreases to a floor, indicating that there exists an optimal transmitting power $P_{t}$ of the SU-Tx for achieving the best performance.

**Figure 3:**
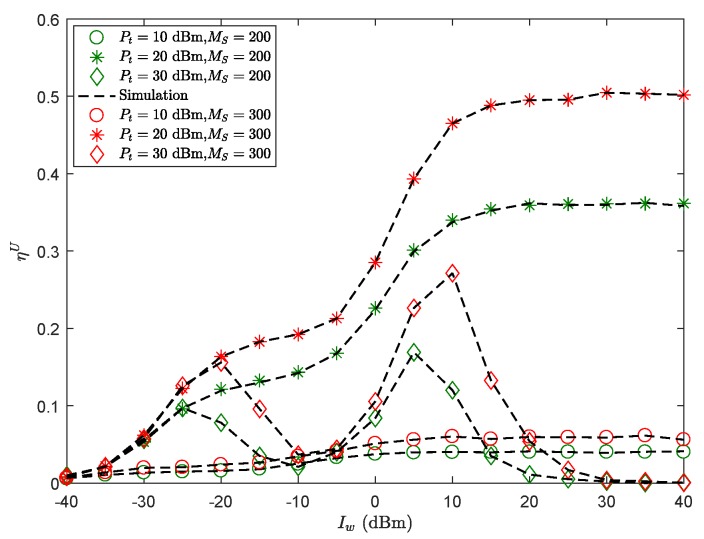
$\eta^{U}$ versus $I_{w}$ with $\lambda_{E}=0.001$ $nodes/m^{2}$, *r* = 60 m, $\theta_{S}=\frac{\pi }{3}$, $R_{m}=4.5$ BPCU, $R_{m}^{s}=1$ BPCU, $\delta_{0}=0.1$, β = $\frac{1}{141.4}$.

Figure 3 presents $\eta^{U}$ of the secondary sensor node versus $I_{w}$ witha different transmit power $P_{t}$. As shown in figure, the ST performance is not always improved with expanding $I_{w}$. This is fairly straightforward because there is a tradeoff between reliability and security caused by $I_{w}$. In addition, the impacts of $P_{t}$ on ST performance have similar trends and cause as $I_{w}$. Besides, we found that when the transmit power $P_{t}$ = 10 dBm, the ST shows a double peak with $I_{w}$, which is mainly due to LOS and NLOS links. Furthermore, with the improving gain of SU-Tx beamforming, the ST would be improved. This can be explained by the high gain antenna improving the receive performance of the secondary sensor node, and increasing the probability of connection; at the same time, it may increase the risk of information leakage.

**Figure 4:**
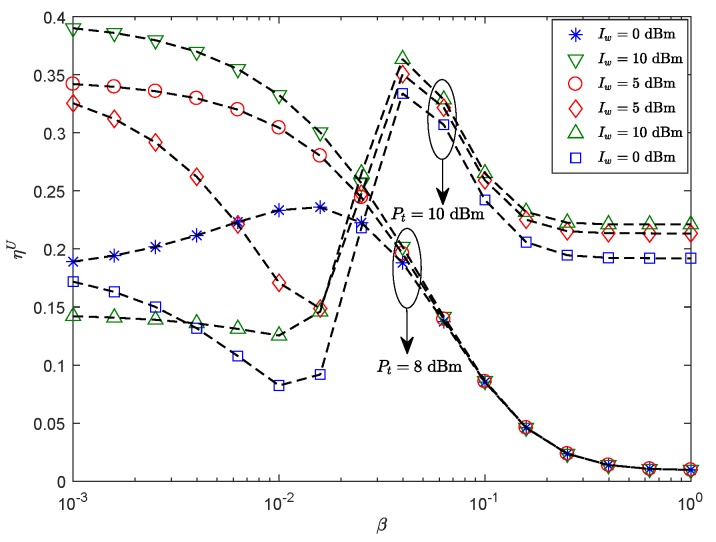
$\eta^{U}$ versus β with $\lambda_{E}=0.001$ $nodes/m^{2}$, *r* = 60 m, $\theta_{S}=\frac{\pi }{3}$, $R_{m}=4.5$ BPCU, $R_{m}^{s}=1$ BPCU, $\delta_{0}=0.1$, $m_{S}=0.1$ and $M_{S}=200$.

Figure 4 plots the effects of $\eta^{U}$ of the secondary sensor node versus β with the different $P_{t}$ and $I_{w}$. We can see that $\eta^{U}$ decreases with increasing β for a lower $P_{t}$. This is because the path loss of mmWave is high; with the increase of β, the ST of the secondary sensor node decreases gradually. Yet, given a high $P_{t}$, increasing β does not result in a strict decrease in ST of the mmWave networks under the constraint of the $I_{w}$. Actually, there exists an optimal $\beta^{*}$, so that a maximum ST can be obtained. NLOS links dominate the mmWave sensor network when β just reaches the optimum point, using multipath signals to maximize ST of secondary sensor nodes. However, when the environment is full of physical barriers, the probability of information reaching the secondary sensor node decreases and the ST decreases until it saturates. This demonstrates that the blockages can also be used to improve the system performance by reasonably setting parameters according to the actual situation in random cognitive mmWave sensor networks.

**Figure 5:**
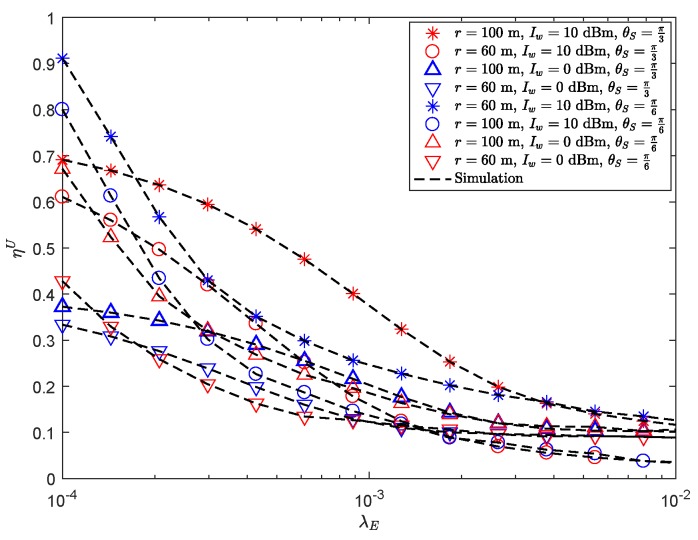
$\eta^{U}$ versus $\lambda_{E}$ with $P_{t}$ = 30 dBm, $R_{m}=4.5$ BPCU, $R_{m}^{s}=1$ BPCU, β = $\frac{1}{141.4}$, $\delta_{0}=0.1$, $m_{S}=0.1$ and $M_{S}=200$.

Figure 5 presents the effects of $\eta^{U}$ of the secondary sensor node versus $\lambda_{E}$ with the different $I_{w}$ and $\theta_{S}$. It is observed that $\eta^{U}$ decreases with the increasing $\lambda_{E}$, since the wiretapping ability of eavesdroppers increases when $\lambda_{E}$ is large. We can also see that under the same conditions, the larger the sector guard zone, the better $\eta^{U}$, because the large sector guard zone helps to improve the secrecy performance of the system. Moreover, when $\lambda_{E}$ is low, the performance of the system with large $I_{w}$ is better than that with small $I_{w}$. When $\lambda_{E}$ is high, the performance of the system with small $I_{w}$ is better than that of the system with large $I_{w}$. The reason for this trend is that the larger $I_{w}$ will lead to worse secrecy performance when increasing $\lambda_{E}$. It demonstrates that sector guard zone and $I_{w}$ play an important role in the performance of secondary sensor node. In addition, with the improvement of the directionality of the beamforming of the SU-Tx, $\eta^{U}$ would be improved. This can be explained by the fact that the high gain narrow beam antenna reduces the information leakage, improves the receive performance of the SU-Rx, and increases the system reliability. However, we can also see that with the increasing of $\lambda_{E}$, the wiretapping ability of eavesdroppers increases, and the narrow beam would lead to the rapid degradation of system performance. This is because the eavesdroppers may be in the beam, which has a fatal impact on the security of the system considered.

## 5. Conclusions

In this paper, we have studied the secrecy performance of the secondary sensor network under the interference temperature constraint of a primary sensor node. Specifically, stochastic geometry-based techniques were adopted for modeling both the locations of sensor nodes and of the eavesdroppers in the networks considered. We considered a special case, where the eavesdroppers and the primary sensor node may be in the secondary network signal beam. Combining the mmWave characteristics, the exact analysis on ST of the secondary sensor node was conducted, and an asymptotic result of ST was further provided. Our results showed that the interference temperature constraint of the primary network can be used to balance the secrecy performance of the secondary network. Moreover, blockages were beneficial to improve the ST of the secondary sensor node under certain conditions. In future works, complex scenarios such as imperfect CSI, base-station (BS) cooperation and nonorthogonal multiple access (NOMA) will be considered. In addition, combining the results presented in this paper with UAV, the secrecy transmission capability may be analyzed.

## Appendix A

Based on Equation (10), $\Psi_{1}$ is calculated in detail. Applying the probability generating function, polar coordinates and [37] (Equation (3.351.1)), we can formulate

(A1) $$\begin{array}{ll} \Psi_{1} & =\mathrm{Pr}\left\{\max_{E\in \phi_{{}_{E}}^{L}}\left(\frac{M_{S}P_{t}{\left|h_{SE}\right|}^{2}L\left(r_{E}\right)}{\sigma_{E}^{2}}\right)<\varepsilon_{2}\right\}=E\left\{\prod_{E\in \phi_{{}_{E}}^{L}}\mathrm{Pr}\left({\left|h_{SE}\right|}^{2}<\frac{\sigma_{E}^{2}\varepsilon_{2}r_{E}^{\alpha_{L}}}{P_{t}C_{L}M_{S}}\right)\right\}\\ & =\exp\left(\;\;\;-\theta_{s}\lambda_{E}\int_{r}^{\infty }\left(1-\frac{\Upsilon \left(N_{L},\frac{\sigma_{E}^{2}\varepsilon_{2}r_{E}^{\alpha_{L}}N_{L}}{P_{t}C_{L}M_{S}}\right)}{\Gamma \left(N_{L}\right)}\right)e^{-\beta r_{{}_{E}}}r_{E}dr_{E}\right)\\ & =\;\exp\;\;\left(\;\;\;\;-\theta_{s}\lambda_{E}\;\;\left(\;\;\frac{\Gamma \left(2,\beta r\right)}{\beta^{2}}\;\;-\;\;\;\;\sum_{n=0}^{\infty }\;\;\;\;\frac{{\left(-1\right)}^{n}{\left(\frac{\sigma_{E}^{2}\varepsilon_{2}N_{L}}{P_{t}C_{L}M_{S}}\right)}^{\;\;N_{L}\;+\;n}\;\;\Gamma \left(\alpha_{L}\left(\;N_{L}\;+\;n\right)+2,\beta r\right)}{n!\left(N_{L}+n\right)\Gamma \left(N_{L}\right)\beta^{\alpha_{L}\left(N_{L}+n\right)+2}}\;\;\right)\;\;\right) \end{array}.$$

Following a procedure similar to that used for obtaining $\Psi_{1}$, $\Psi_{2}$ can be obtained. For the sake of brevity, the calculation procedure of $\Psi_{2}$ is omitted.

Substituting $\Psi_{1}$ and $\Psi_{2}$ into Equation (10) and after some mathematical manipulations, $F_{y}\left(\varepsilon_{2}\right)$ can be expressed as

(A2) $$\begin{array}{ll} F_{y}\left(\varepsilon_{2}\right) & =\exp\left(-\theta_{s}\lambda_{E}\left(\left(\frac{\left(N_{N}-1\right)!}{\Gamma \left(N_{N}\right)}\right)\sum_{m=0}^{N_{N}-1}\frac{{\left(\frac{\sigma_{E}^{2}\varepsilon_{2}N_{N}}{P_{t}C_{N}M_{S}}\right)}^{m}}{m!}\times \frac{\Gamma \left(\frac{m\alpha_{N}+2}{\alpha_{N}},\frac{\sigma_{E}^{2}\varepsilon_{2}r^{\alpha_{N}}N_{N}}{P_{t}C_{N}M_{S}}\right)}{\alpha_{N}{\left(\frac{\sigma_{E}^{2}\varepsilon_{2}N_{N}}{P_{t}C_{N}M_{S}}\right)}^{\frac{m\alpha_{N}+2}{\alpha_{N}}}}\right.\right.\;\\ & +\left.\left.\sum_{n=0}^{\infty }\frac{{\left(-1\right)}^{n}{\left(\frac{\sigma_{E}^{2}\varepsilon_{2}N_{N}}{P_{t}C_{N}M_{S}}\right)}^{N_{N}+n}}{n!\left(N_{N}+n\right)\Gamma \left(N_{N}\right)}\frac{\Gamma \left(\alpha_{N}\left(N_{N}+n\right)+2,\beta r\right)}{\beta^{\alpha_{N}\left(N_{N}+n\right)+2}}-\sum_{n=0}^{\infty }\frac{{\left(-1\right)}^{n}{\left(\frac{\sigma_{E}^{2}\varepsilon_{2}N_{L}}{P_{t}C_{L}M_{S}}\right)}^{N_{L}+n}}{n!\left(N_{L}+n\right)\Gamma \left(N_{L}\right)}\frac{\Gamma \left(\alpha_{L}\left(N_{L}+n\right)+2,\beta r\right)}{\beta^{\alpha_{L}\left(N_{L}+n\right)+2}}\right)\right) \end{array}.$$

Similarly, $F_{v}\left(\varepsilon_{1}\right)$ is obtained for Equation (11), which can be expressed as

(A3) $$\begin{array}{ll} F_{v}\left(\varepsilon_{1}\right) & =\frac{2}{R_{U}^{2}}\left(\sum_{i=0}^{\infty }\frac{{\left(-1\right)}^{i}{\left(A\varepsilon_{1}N_{L}\right)}^{N_{L}+i}}{i!\left(N_{L}+i\right)\Gamma \left(N_{L}\right){\left(P_{t}M_{S}C_{L}\right)}^{N_{L}+i}}\times \frac{\Upsilon \left(\alpha_{L}\left(N_{L}+i\right)+2,\beta R_{U}\right)}{\beta^{\alpha_{L}\left(N_{L}+i\right)+2}}\right.\;\\ & +\left.\sum_{j=0}^{\infty }\frac{{\left(-1\right)}^{j}{\left(A\varepsilon_{1}N_{N}\right)}^{N_{N}+j}}{j!\left(N_{N}+j\right)\Gamma \left(N_{N}\right){\left(P_{t}M_{S}C_{N}\right)}^{N_{N}+j}}\left.\left(\frac{{\left(R_{U}\right)}^{\alpha_{N}\left(N_{N}+j\right)+2}}{\alpha_{N}\left(N_{N}+j\right)+2}-\frac{\Upsilon \left(\alpha_{N}\left(N_{N}+j\right)+2,\beta R_{U}\right)}{\beta^{\alpha_{N}\left(N_{N}+j\right)+2}}\right.\right)\right) \end{array}.$$

Substituting $F_{y}\left(\varepsilon_{2}\right)$ and $F_{v}\left(\varepsilon_{1}\right)$ into Equation (9), the proof is completed.

## Appendix B

Based on Equation (14), let us now turn our attention on $\Psi_{3}$. Using the probability generating function, polar coordinates and [37] (Equation (3.351.1)), we can formulate

(A4) $$\begin{array}{ll} \Psi_{3} & =\mathrm{Pr}\left\{\max_{E\in \phi_{{}_{E}}^{L}}\left(\frac{I_{w}{\left|h_{SE}\right|}^{2}L\left(r_{E}\right)}{{\left|h_{SP}\right|}^{2}L\left(r_{SP}\right)\sigma_{E}^{2}}\right)<\varepsilon_{2}\right\}\;\\ & =\;\;\exp\;\;\;\left(\;\;\;-\theta_{s}\lambda_{E}\;\;\left(\;\;\frac{\Gamma \left(2,\beta r\right)}{\beta^{2}}\;\;-\;\;\;\;\sum_{n=0}^{\infty }\;\;\;\;\frac{{\left(\;-\;1\right)}^{n}{\left(\frac{\sigma_{E}^{2}\varepsilon_{2}xN_{L}}{I_{w}C_{L}}\right)}^{\;\;N_{L}\;+\;n}\;\;\Gamma \left(\alpha_{L}\left(\;N_{L}\;+\;n\right)+2,\beta r\right)}{n!\left(N_{L}+n\right)\Gamma \left(N_{L}\right)\beta^{\alpha_{L}\left(N_{L}+n\right)+2}}\;\;\right)\;\;\right) \end{array}.$$

Following a procedure similar to that used for obtaining $\Psi_{3}$, $\Psi_{4}$ can be obtained. For the sake of brevity, the calculation procedure of $\Psi_{4}$ is omitted.

Upon substituting $\Psi_{3}$ and $\Psi_{4}$ into Equation (14), as well as after some mathematical manipulations, the expressions for $F_{z}\left(\varepsilon_{2}\right)$ and $F_{u}\left(\varepsilon_{1}\right)$ are expressed as

(A5) $$\begin{array}{ll} F_{z}\left(\varepsilon_{2}\right) & =\exp\left(-\right.\theta_{s}\lambda_{E}\left(\left(\frac{\left(N_{N}-1\right)!}{\Gamma \left(N_{N}\right)}\right)\sum_{m=0}^{N_{N}-1}\frac{{\left(\frac{\sigma_{E}^{2}\varepsilon_{2}xN_{N}}{I_{w}C_{N}}\right)}^{m}}{m!}\times \frac{\Gamma \left(\frac{m\alpha_{N}+2}{\alpha_{N}},\frac{\sigma_{E}^{2}\varepsilon_{2}xr^{\alpha_{N}}N_{N}}{I_{w}C_{N}}\right)}{\alpha_{N}{\left(\frac{\sigma_{E}^{2}\varepsilon_{2}xN_{N}}{I_{w}C_{N}}\right)}^{\frac{m\alpha_{N}+2}{\alpha_{N}}}}\right.\;\\ & +\left.\left.\sum_{n=0}^{\infty }\frac{{\left(-1\right)}^{n}{\left(\frac{\sigma_{E}^{2}\varepsilon_{2}xN_{N}}{I_{w}C_{N}}\right)}^{N_{N}+n}}{n!\left(N_{N}+n\right)\Gamma \left(N_{N}\right)}\frac{\Gamma \left(\alpha_{N}\left(N_{N}+n\right)+2,\beta r\right)}{\beta^{\alpha_{N}\left(N_{N}+n\right)+2}}-\sum_{n=0}^{\infty }\frac{{\left(-1\right)}^{n}{\left(\frac{\sigma_{E}^{2}\varepsilon_{2}xN_{L}}{I_{w}C_{L}}\right)}^{N_{L}+n}}{n!\left(N_{L}+n\right)\Gamma \left(N_{L}\right)}\frac{\Gamma \left(\alpha_{L}\left(N_{L}+n\right)+2,\beta r\right)}{\beta^{\alpha_{L}\left(N_{L}+n\right)+2}}\right)\right) \end{array}.$$

(A6) $$\begin{array}{ll} F_{u}\left(\varepsilon_{1}\right) & =\frac{2}{R_{U}^{2}}\left(\sum_{i=0}^{\infty }\frac{{\left(-1\right)}^{i}{\left(Ax\varepsilon_{1}N_{L}\right)}^{N_{L}+i}}{i!\left(N_{L}+i\right)\Gamma \left(N_{L}\right){\left(I_{w}C_{L}\right)}^{N_{L}+i}}\times \frac{\Upsilon \left(\alpha_{L}\left(N_{L}+i\right)+2,\beta R_{U}\right)}{\beta^{\alpha_{L}\left(N_{L}+i\right)+2}}\right.\\ & +\left.\sum_{j=0}^{\infty }\frac{{\left(-1\right)}^{j}{\left(Ax\varepsilon_{1}N_{N}\right)}^{N_{N}+j}}{j!\left(N_{N}+j\right)\Gamma \left(N_{N}\right){\left(I_{w}C_{N}\right)}^{N_{N}+i}}\left.\left(\frac{{\left(R_{U}\right)}^{\alpha_{N}\left(N_{N}+j\right)+2}}{\alpha_{N}\left(N_{N}+j\right)+2}-\frac{\Upsilon \left(\alpha_{N}\left(N_{N}+j\right)+2,\beta R_{U}\right)}{\beta^{\alpha_{N}\left(N_{N}+j\right)+2}}\right.\right)\right) \end{array}.$$

Upon substituting $F_{z}\left(\varepsilon_{2}\right)$ and $F_{u}\left(\varepsilon_{1}\right)$ into Equation (13), $Q_{2}$ is given by Equation (16).
